# Engineering NK-CAR.19 cells with the IL-15/IL-15Rα complex improved proliferation and anti-tumor effect *in vivo*

**DOI:** 10.3389/fimmu.2023.1226518

**Published:** 2023-09-25

**Authors:** Renata Nacasaki Silvestre, Jiri Eitler, Julia Teixeira Cottas de Azevedo, Mariane Cariati Tirapelle, Daianne Maciely Carvalho Fantacini, Lucas Eduardo Botelho de Souza, Kamilla Swiech, Dimas Tadeu Covas, Rodrigo T. Calado, Paola Ortiz Montero, Kelen Cristina Ribeiro Malmegrim, Marxa L. Figueiredo, Torsten Tonn, Virginia Picanço-Castro

**Affiliations:** ^1^ Center for Cell-based Therapy CTC, Regional Blood Center of Ribeirão Preto, University of São Paulo, Ribeirão Preto, SP, Brazil; ^2^ Experimental Transfusion Medicine, Faculty of Medicine Carl Gustav Carus, Dresden University of Technology, Dresden, Germany; ^3^ Institute for Transfusion Medicine, German Red Cross Blood Donation Service North-East, Dresden, Germany; ^4^ Department of Clinical Analyses, Toxicology and Food Science, School of Pharmaceutical Sciences of Ribeirão Preto, University of São Paulo, Ribeirão Preto, Brazil; ^5^ Department of Basic Medical Sciences, Purdue University, West Lafayette, IN, United States; ^6^ German Cancer Consortium (DKTK), Partner Site Dresden, Dresden, Germany

**Keywords:** CAR-NK cells, NK-92, adoptive cell therapy, IL-15, IL-15 receptor, B-cell malignances, allogeneic therapy

## Abstract

**Introduction:**

Natural killer 92 (NK-92) cells are an attractive therapeutic approach as alternative chimeric antigen receptor (CAR) carriers, different from T cells, once they can be used in the allogeneic setting. The modest *in vivo* outcomes observed with NK-92 cells continue to present hurdles in successfully translating NK-92 cell therapies into clinical applications. Adoptive transfer of CAR-NK-92 cells holds out the promise of therapeutic benefit at a lower rate of adverse events due to the absence of GvHD and cytokine release syndrome. However, it has not achieved breakthrough clinical results yet, and further improvement of CAR-NK-92 cells is necessary.

**Methods:**

In this study, we conducted a comparative analysis between CD19-targeted CAR (CAR.19) co-expressing IL-15 (CAR.19-IL15) with IL-15/IL-15Rα (CAR.19-IL15/IL15Rα) to promote NK cell proliferation, activation, and cytotoxic activity against B-cell leukemia. CAR constructs were cloned into lentiviral vector and transduced into NK-92 cell line. Potency of CAR-NK cells were assessed against CD19-expressing cell lines NALM-6 or Raji in vitro and in vivo in a murine model. Tumor burden was measured by bioluminescence.

**Results:**

We demonstrated that a fourth- generation CD19-targeted CAR (CAR.19) co-expressing IL-15 linked to its receptor IL-15/IL-15Rα (CAR.19-IL-15/IL-15Rα) significantly enhanced NK-92 cell proliferation, proinflammatory cytokine secretion, and cytotoxic activity against B-cell cancer cell lines *in vitro* and in a xenograft mouse model.

**Conclusion:**

Together with the results of the systematic analysis of the transcriptome of activated NK-92 CAR variants, this supports the notion that IL-15/IL-15Rα comprising fourth-generation CARs may overcome the limitations of NK-92 cell-based targeted tumor therapies in vivo by providing the necessary growth and activation signals.

## 1 Introduction

Natural killer-92 (NK-92) cells are an attractive alternative to chimeric antigen receptor (CAR)-T cells. CAR-NK therapy is less likely to cause cytokine release syndrome (CRS) due to lower levels of pro-inflammatory cytokines and absence of interleukin-6 (IL-6) expression, which is reported to be a hallmark of CRS. CAR-NK cells can be used in a non-autologous manner due to the absence of GvHD reactivity, thus serving as universal off-the-shelf cell products that are readily available for clinical applications, as their culture expansion and cryopreservation methods improves (1). Therefore, CAR-NK therapy is generally considered to be safer than CAR-T therapy (2). Allogeneic CAR-NK cells can be generated, expanded, cryopreserved, and supplied to patients on demand at significantly reduced manufacturing costs.

Despite their potential therapeutic value, the translation of NK-92 cells into clinical settings faces several challenges. These challenges include limited expansion capacity both *in vitro* and *in vivo* given high production costs due to the addition of exogenous cytokines, alternative expansion agents, longer expansion times, and higher cell dose required per patient. Although initial results from clinical trials with CAR-NK-92 cells in hematologic malignancies are encouraging (3), long-term therapeutic efficacy has not been demonstrated. To improve proliferative potential and efficacy, fourth generation CAR constructs have been generated to provide additional prosurvival signals to NK cells. The use of IL-15 transgenic expression is a possible strategy (4).

IL-15 has been suggested to allow the expansion of NK cells *in vivo* (4). IL-15 is a cytokine, physiologically released by dendritic cells, monocytes, and macrophages, that plays a key role in the development, homeostasis, activation, and survival of T, natural killer (NK), and NK-T cells (5). IL-15 cytokine has at least three functional forms: (i) a soluble monomeric IL-15 (sIL-15), (ii) soluble IL-15/IL-15Rα complex, and (iii) membrane-bound IL-15/IL-15Rα (6–8). IL-2 as well as IL-15 are members of the γ chain family of cytokines and are responsible for NK activation and proliferation of NK cells. Both cytokines share the γc and IL2/IL15Rβ chains. Additionally, they have specific subunits, IL-2RC or IL-15Rα, which form the IL-2 and/or IL-15 receptors, respectively (9).

IL-15 exhibits its effects through two distinct mechanisms: trans presentation and cis presentation. In trans presentation, IL-15 forms a complex with its high-affinity receptor, IL-15Rα, on the surface of a specific cell (referred to as the presenting cell). This complex is then recognized by the β and γ receptor chains located on the surface of another cell (referred to as the responding cell). Conversely, in cis presentation, IL-15 binds directly to the β and γ receptor complex on the same cell’s surface that is producing IL-15. Notably, the cis presentation mechanism can trigger the activation and proliferation of the very cell generating IL-15. Both trans and cis presentations of IL-15 play pivotal roles in regulating immune responses. The contributions of these mechanisms are context-dependent and can vary based on the specific cell types involved (10–12).

Although the IL-15/IL-15Rα complex has greater potency, bioavailability, and stability compared to soluble monomeric IL-15, when administered to mice and humans (13–16), the soluble monomeric IL-15 presentation has been the most common form incorporated in the expression of vectors used in CAR-NK cells to improve NK potency *in vivo*, including in some recent clinical trials (3, 4).

In this context, our objective was to systematically compare CAR constructs that have potential for clinical use, and incorporate different forms of recombinant IL-15, in order to assess their potential for improving NK cell activation and proliferation. In our study, using NK-92 as a model, we investigated the different effects of soluble IL15, and a membrane bound protein consisting of human IL-15 and human IL-15Rα fused by flexible linkers (IL-15/IL-15Ra) to overcome NK potency and expansion capacity limitations *in vitro* and in a murine xenograft model for CD19 hematologic malignancies. We have generated two fourth generation CD19-targeted CARs (CAR.19) co-expressing either soluble IL-15 (CAR.19-IL-15) or IL-15/IL-15Rα (CAR.19-IL-15/IL-15Rα), and compared them with a second-generation CAR.19 for their capacity to promote NK cell survival, activation, proliferation, *in vitro* cytotoxic activity against B-cell lines, as well as *in vivo* therapeutic efficacy. Moreover, we have evaluated the transcriptional profiles of NK-92-CAR.19 cell variants to elucidate the pathways involved in IL-15 and IL-15/IL-15Rα signaling.

## 2 Methods

### 2.1 Cells and culture medium

The Natural Killer 92 (NK-92) cell line, as described by Gong et al. (1994) (17) and obtained from ATCC (CRL-2407™), was cultured using serum-free X-VIVO 10 media (Lonza, Cologne, Germany). containing 5% human heat inactivated AB plasma (Brazilian Blood Donation Service of the Regional Blood Center of Ribeirão Preto), supplemented with 500 IU/ml IL-2 (Clinigen), as previously described (18, 19). Human leukemia cell lines K562, Raji, and NALM-6 were cultured in RPMI medium (ThermoFisher Scientific) supplemented with 10% Fetal Bovine Serum (FBS). All cell lines tested negative for mycoplasma using the MycoAlertPLUS Mycoplasma Detection Kit (Lonza) (reference value< 1.0).

### 2.2 STR profiling

All cell lines were authenticated by short tandem repeat (STR) analysis. This methodology uniquely identifies human cell lines derived from the tissue of a single individual. A minimum of eight major STR loci are required to identify a human cell line. STR loci were amplified using specific primers for each loci with a PCR amplification kit (Sigma-Aldrich) (detecting D5S818, D7S820, CSF1PO, vWA, D16S539, TPOX, THO, and D13S317). Electrophoretic analysis with polyacrylamide gel was performed and stained with silver nitrate. The following STR profile were analyzed: NK-92 cell (ATCC CRL-2407) (CSF1PO: 11,12; D13S317: 9,12; D16S539: 11,12; D5S818: 12,13; D7S820: 10, 11; THO: 6, 9.3; TPOX: 8; vWA: 18), Raji cell (ATCC CCL-86) (CSF1PO: 10, 12; D13S317: 13; D16S539: 8, 11; D5S818: 10, 13; D7S820: 10; THO1: 6, 7; TPOX:8,13; vWA: 16, 19), K562 cell line (ATCC CCL-243) (CSF1PO: 9,10; D13S317: 8; D16S539: 11,12; D5S818: 11,12; D7S820: 9, 11; THO: 9.3; TPOX: 8,9; vWA: 16), HEK293T: (CSF1PO: 11, 12; D13S317: 12, 14; D16S539: 9, 13; D5S818: 8, 9; D7S820: 11; THO1: 7, 9.3; TPOX: 11; vWA: 16, 19). Nalm-6 cell line (CSF1PO: 12; D13S317: 9,12,8,11; D16S539: 10,11; D5S818: 11,12; D7S820: 8,10,9; THO1: 8, 9; TPOX:8,10; vWA: 15,16). The values found were in accordance with the description of cell lines from the ATCC and DSMZ bank.

### 2.3 Construction of lentiviral vectors and transduction of NK-92 cells

The lentiviral vectors encoding anti-CD19 CAR (CAR.19) were constructed based on a previously described vector (20). CAR.19 consists of murine anti-CD19 single chain fragment variable (scFv) from the antibody clone HD-37, CD8 hinge and transmembrane region, 4-1BB costimulatory domain, and CD3ζ cytoplasmic region. CAR.19-IL-15 and CAR.19-IL-15/IL-15Rα additionally contain IL-15 or IL-15/IL-15Rα separated by a T2A peptide. IL-15 was linked to IL-15Rα as previously described (21). Lentiviral production was generated by the transient cotransfection of HEK 293 T cells with the three-plasmid system previously described (20). Lentiviral stock titers were determined in a K562 cell line. NK-92 cells were transduced in the presence of 8 µg/ml polybrene and 1000 IU/mL IL-2 for 5 hours at 37°C.

### 2.4 Enrichment of NK-92-CAR cells

NK-92-CAR cells were enriched by positive selection using magnetic beads. Cells were incubated with biotin-SP-conjugated anti-mouse F(ab’)2 antibodies (Jackson Immunoresearch) followed by incubation with anti-biotin MicroBeads (Miltenyi Biotec). Separation was performed using MACS separation columns (Miltenyi Biotec) according to the manufacturer’s instructions. The procedure was repeated 7 days after the initial selection.

### 2.5 Flow cytometry

For flow cytometry, NK-92 cells were stained with monoclonal antibodies specific for CD56, CD3, CD45, CD28, CD16, CD11a, CD2, NKG2D, NKp30, NKp46, DNAM-1, CD95 (BD Bioscience) for 30 minutes on ice. CAR expression on the surface of NK-92 cells was detected with an anti-mouse F(ab’)2 antibody (Jackson Immunoresearch). For checkpoint molecules, antibodies specific for PD-1, LAG-3 and TIM-3 (BD Biosciences) were used. Dead cells were excluded using 7-AAD viability dye (BD Biosciences). Samples were acquired using an LSR-Fortessa cell analyzer (BD Biosciences), and data were analyzed using FlowJo 10 software (BD Biosciences).

### 2.6 Cytotoxicity assays

The cytotoxicity of NK-92 cells against established cancer B-cell lines was analyzed by a flow cytometry-based assay as previously described (20). Briefly, target cells were labeled with PKH67 (Sigma-Aldrich) and incubated with effector cells at various effector to target (E:T) ratios (2:1 and 10:1) for 5 hours at 37°C. 7-AAD solution was added to each sample prior to flow cytometric analysis. Cells were acquired using LSR-Fortessa cell analyzer (BD Biosciences). Dead target cells were identified as double positive for PKH67 and 7-AAD. Cell cytotoxicity was analyzed by measuring the difference in the percentage of living cells at 0 and 5 hours after cocultivation. The percentage of cell death (% cytotoxicity) was calculated using the following equation:


$$Cytotoxicity\left(\%\right)=100-\left(\frac{\%\;live\;B\;cells\;\left(5h\right)}{\%\;live\;B\;cells\;\left(0h\right)}\right)*100$$

### 2.7 Cytokine release assay

For the cytokine release assay, 2.5x 10^5^ NK-92 cells were co-cultured or not with 0.25x10^5^ cancer cells at 37°C for 5 hours. Supernatants were harvested and IL-15, IFN-γ and TNFα were measured by multiplex assay using the MILLIPLEX MAP Human CD8+ T-Cell magnetic Bead Panel kit and MILLIPLEX Human Cytokine/Chemokine/Growth factor Panel A (Merck-Millipore). The MAGPIX ® System (Luminex Corporation) was used for data analysis according to manufacturer’s specifications. Cytokine concentrations were quantified using the Milliplex® Analyst software.

### 2.8 RNAseq

NK-92 cells were cocultured with Raji cell at 10:1 ratio (E:T) for 24h at 37°C, enough time to eliminate target cells. Total RNA from NK-92 cells was isolated from two biological replicates. In short, RNA was isolated using RNeasy kit (Qiagen) according to manufacturer’s protocol. Poly(A) RNA sequencing library was prepared following Illumina’s TruSeq-stranded-mRNA protocol and conducted by LC Sciences (Houston, TX, USA). Poly(A) tail-containing mRNAs were purified using oligo-dT. Briefly, mRNA was extracted using magnetic beads with two rounds of purification, and then fragmented using a divalent cation buffer at an elevated temperature. Quality control analysis and quantification of the sequencing library were performed using an Agilent Technologies 2100 Bioanalyzer High Sensitivity DNA Chip. Paired-ended sequencing was performed on Illumina’s NovaSeq 6000. For transcript assembly and estimating transcript expression levels, the software is described by Figueiredo et al. (2020) (22). For differential expression analysis of mRNAs, StringTie was used by calculating fragments per kilobase million (FPKM). The differentially expressed mRNAs were selected with log2 (fold change) > 1.5 or log2 (fold change)< -1.5 and with statistical significance (p< 0.05) by edgeR. The datasets generated in this study will be submitted to the Gene Expression Omnibus (GEO) repository upon acceptance with the assistance of the Purdue Bioinformatics Core.

### 2.9 *In vivo* murine B-cell lymphoma model

Eight to 13-week-old male NSG mice were injected intravenously with 2 × 10^4^ Raji/Luc cells at day 0. On days 4, 7, 10, 12, 15, and 19, after tumor cell inoculation, 7 × 10^6^ NK-92-CAR.19 or parental NK-92 cells were injected intravenously. Disease development was monitored using an *in vivo* imaging system (IVIS, Perkin Elmer) after intraperitoneal injection of 150 mg/kg of D-luciferin (Perkin Elmer). For *in vivo* experiments, all applicable guidelines for animal care and use were followed and animal experiments were approved by the responsible government committee in Brazil (n. 124/2017).

### 2.10 Statistical analysis

All statistical analyses were performed using Graphpad Prism 8.0 software. The statistical tests used are indicated in the figure legends. A P value<0.05 was considered statistically significant. ****P<0.0001; ***P<0.001; **P<0.01; *P<0.05; ns (not significant) P ≥ 0.05.

## 3 Results

### 3.1 Generation of NK-92-CAR.19-IL-15 and NK-92-CAR.19-IL-15/IL-15Rα cells

**Figure 1:**
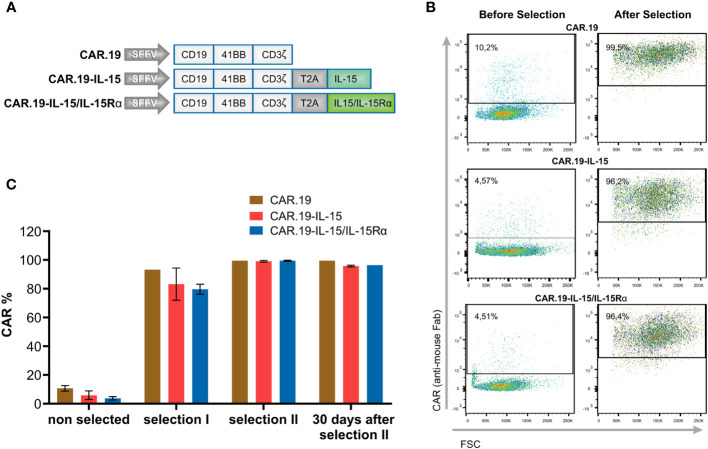
Generation of NK-92-CAR.19-IL-15/IL15Rα cells. **(A)** Scheme of CAR constructs targeting CD19 (CAR.19) under the control of spleen focus-forming virus (SFFV) promoter. CAR consists of scFv, CD8 hinge and transmembrane region, 41BB costimulatory molecule and CD3ζ signaling molecule. Where indicated, the CAR sequences are followed by a self-cleaving peptide (T2A) and IL-15 or IL-15/IL-15Rα (IL-15 and IL-15Rα fused through a flexible linker). **(B, C)** NK-92 cells were transduced with the described CAR constructs followed by immunomagnetic enrichment in two steps. **(B)** Representative dot plots show flow cytometric analysis of CAR expression on freshly transduced and enriched NK-92-CAR.19 cells. **(C)** The graph shows the frequency of CAR-positive cells before selection, after first and second selection, and 30 days after second selection.

We constructed lentiviral vectors carrying a fourth generation CAR with specificity for the CD19 molecule (CAR.19) and co-expressing soluble IL-15 or IL-15/IL-15Rα molecules. CAR consists of the anti-CD19 specific scFv fragment, CD8 hinge and transmembrane regions, and the intracellular signaling domains of human 4-1BB and the CD3ζ motif in tandem. A 2A self-cleaving peptide sequence was inserted downstream of CAR, followed by IL-15 or IL-15/IL-15Rα molecules (**Figure 1A**). A second-generation CAR.19 without IL-15 cytokine was generated as a control. The CAR sequences were inserted into the self-inactivating lentiviral vector pSEW. VSV-G pseudotyped lentiviral vector particles were generated and used for transduction of human NK-92 cells, resulting in NK-92-CAR.19, NK-92-CAR.19-IL-15, and NK-92-CAR.19-IL-15/IL-15Rα cells. NK-92-CAR cells were enriched by two consecutive rounds of immunomagnetic positive selection. Following the selection process, over 90% of NK-92 cells exhibited positive CAR expression (refer to **Figure 1B**). This outcome led to the establishment of a uniform NK-92-CAR positive cell population. Subsequently, the chosen cells were cultured for a duration of 30 days and consistently maintained a stable expression of CAR (as depicted in **Figure 1C**). After generation and expansion of NK-92-CAR.19 cells, we have characterized immunophenotypically the different engineered NK-92-CAR.19 cells. The expression levels of CD56, CD45, CD95, NKp30, NKp46, NKG2D, CD28, CD2 (LFA-2) and CD11a (LFA-1), were all very similar among the engineered NK-92-CAR.19 and parental NK-92 cells (**Supplementary Figure 1**).

### 3.2 NK-92-CAR.19-IL-15/IL-15Rα cells proliferate independently of IL-2

**Figure 2:**
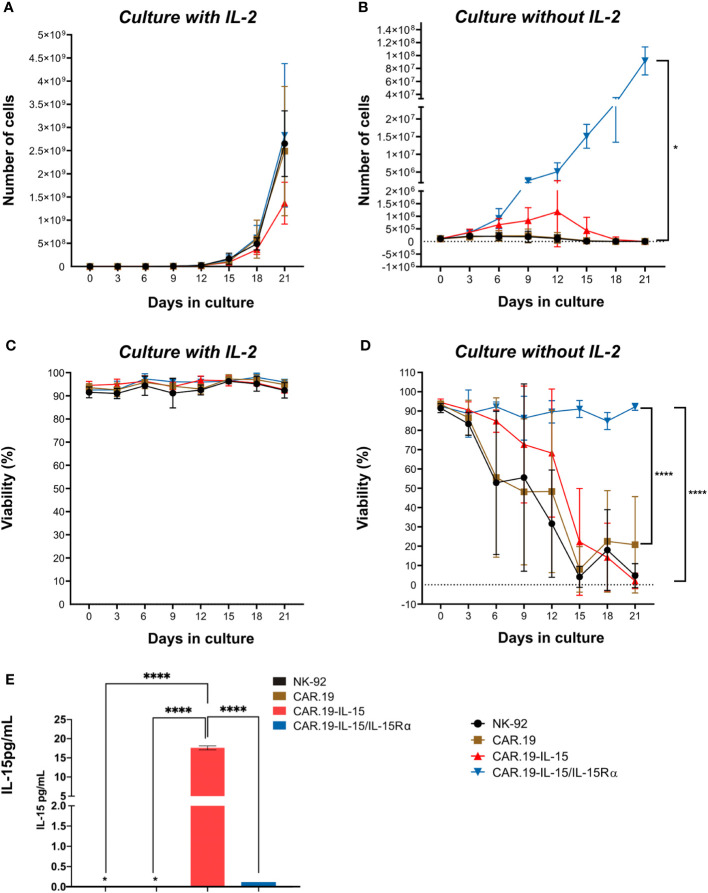
NK-92-CAR.19-IL-15/IL15Rα cells proliferate independently of exogenous IL-2. Proliferation of parental and genetically modified NK-92 cells: NK-92-CAR.19, NK-92-CAR.19-IL-15 and NK-92-CAR.19-IL-15/IL15Rα in X-Vivo 10 supplemented with 5% of human plasma. **(A)** Cells were cultured either in presence of IL-2 (500 UI/mL) or **(B)** in absence of exogenous cytokines. **(C)** Viability of cell culture with cytokines and **(D)** without cytokines. Cell culture was followed over 21 days. Pooled data of two independent experiments (each one performed in duplicates) are shown. **(E)** Quantification of soluble IL-15 in supernatant of NK cell culture by Luminex (n=2). Two-way ANOVA statistical test, Tukey's post-multiple comparison posttest. ****P<0.0001.

Next, we evaluated whether the intrinsic cytokine production by the transgenic cells would be sufficient to replace the addition of exogenous IL-2 in the cultures to allow cell expansion. The NK-92-CAR.19 cells with different vector constructions were cultured in the presence or absence of IL-2 and cell proliferation was evaluated for 21 days. While in the presence of IL-2, all NK-92-CAR.19 versions expanded at an expansion rate typical of parental NK-92 cells and maintained high viability above 90% (**Figures 2A, C**). The withdrawal of IL-2 resulted in an immediate reduction of cell proliferation in parental NK-92 and NK-92-CAR.19 cells and a reduction in cell viability, eventually leading to complete cell death (**Figures 2B, D**). NK-92 cells transduced with a CAR comprising the soluble form of IL-15 were able to proliferate initially at a reduced rate until day 12 of culture despite an overall reduction in viability, before the proliferation finally stopped and the cells died off. In contrast, NK-92-CAR.19-IL-15/IL-15Rα cells successfully expanded throughout the 21-day culture period (**Figure 2B**). Notably, cell viability was maintained between 80-95% during the 21 days of culture without IL-2 (**Figure 2D**). NK-92-CAR.19-IL-15 cells secreted high levels of IL-15 (**Figure 2E**). As expected, only low levels of IL-15 were detected in the supernatant of NK-92-CAR.19-IL-15/I-15Rα cells, because in this case IL-15 is expected to remain bound to its high-affinity Rα receptor on the cell surface and not be secreted.

### 3.3 IL-15 and IL-15/IL-15Rα enhances the antitumor function of NK-92-CAR.19 cells against CD19+ cell lines

**Figure 3:**
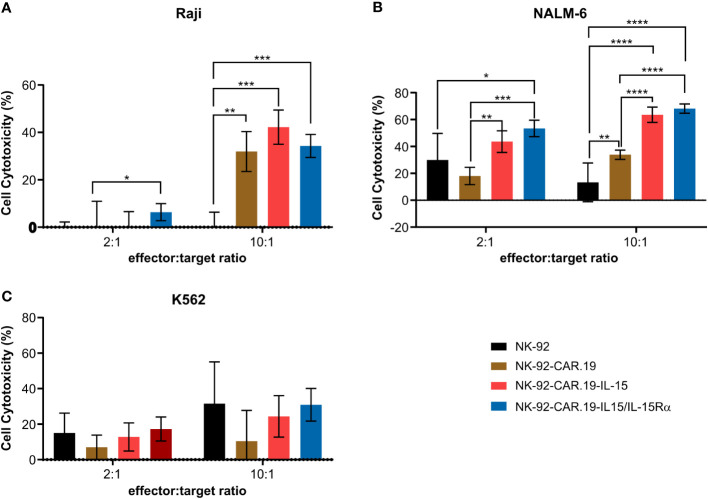
NK-92-CAR.19 cells coexpressing IL-15/Rα enhance CAR-mediated anti-tumor Ncell function. NK-92, NK-92-CAR.19, NK-92-CAR.19-IL-15 and NK-92-CAR.19-IL-15/IL-15Rα cells were co-cultivated with **(A)** Raji **(B)** NALM-6 and **(C)** K562 in 2:1 and 10:1 effector:target cells ratio, for 5 hours. Cytotoxicity was measured by flow cytometry cytotoxicity assay. One-way ANOVA statistical test, Tukey's multiple comparison posttest. ****P < 0.0001; ***P < 0.001; **P < 0.01; *P < 0.05. Mean values + SD are shown. Data pooled from three independent experiments.

We further evaluated the effect of IL-15Rα on the specific cytotoxicity of NK-92-CAR.19 against CD19+ B-cell malignancies. Two CD19-positive cell lines, Raji and NALM-6, and a CD19-negative K562 were used as models. In co-culture assays, all CD19-specific CAR NK-92 cells exhibited enhanced cytotoxicity against NALM-6 and Raji cells compared to unmodified NK-92 cells (**Figure 3**). We did not observe any specific killing of K562 cells by the NK-92-CAR.19 cells. NK-92-CAR.19 cells expressing IL-15 or IL-15/IL-15Rα were significantly more cytotoxic against NALM-6 cells than control NK-92-CAR.19 cells. In order to explain the differences in the results obtained with Raji and NALM6, the expression level of CD19 in these cell lines were evaluated. Variances in the expression of the CD19 antigen between Raji and Nalm-6 cells could indeed result in discrepancies in the recognition and elimination of cancer cells by CAR-NK cells. Raji and Nalm-6 have more than 98% cells positive to CD19 and MFI of Raji is higher than Nalm-6 (**Supplementary Figure 2**). Notably, our findings revealed that Raji cells exhibit higher CD19 expression levels compared to Nalm-6 cells. We can suggest that Raji cells, characterized by their high expression of CD19, are effectively targeted and eliminated by CD19 CAR NK cells. Therefore, any additional enhancement through IL-15/IL-15Rα coexpression might not yield as pronounced results, especially when contrasted with the situation involving low-expression NALM6 CD19 cells. In simpler terms, IL-15/IL-15RA could prove particularly valuable in the context of tumor cells exhibiting minimal levels of the target antigen expression.

### 3.4 IL-15/IL-15Rα expression in NK-92-CAR.19 cells leads to higher secretion of INF-γ and TNF-α after stimulation with Raji cells

**Figure 4:**
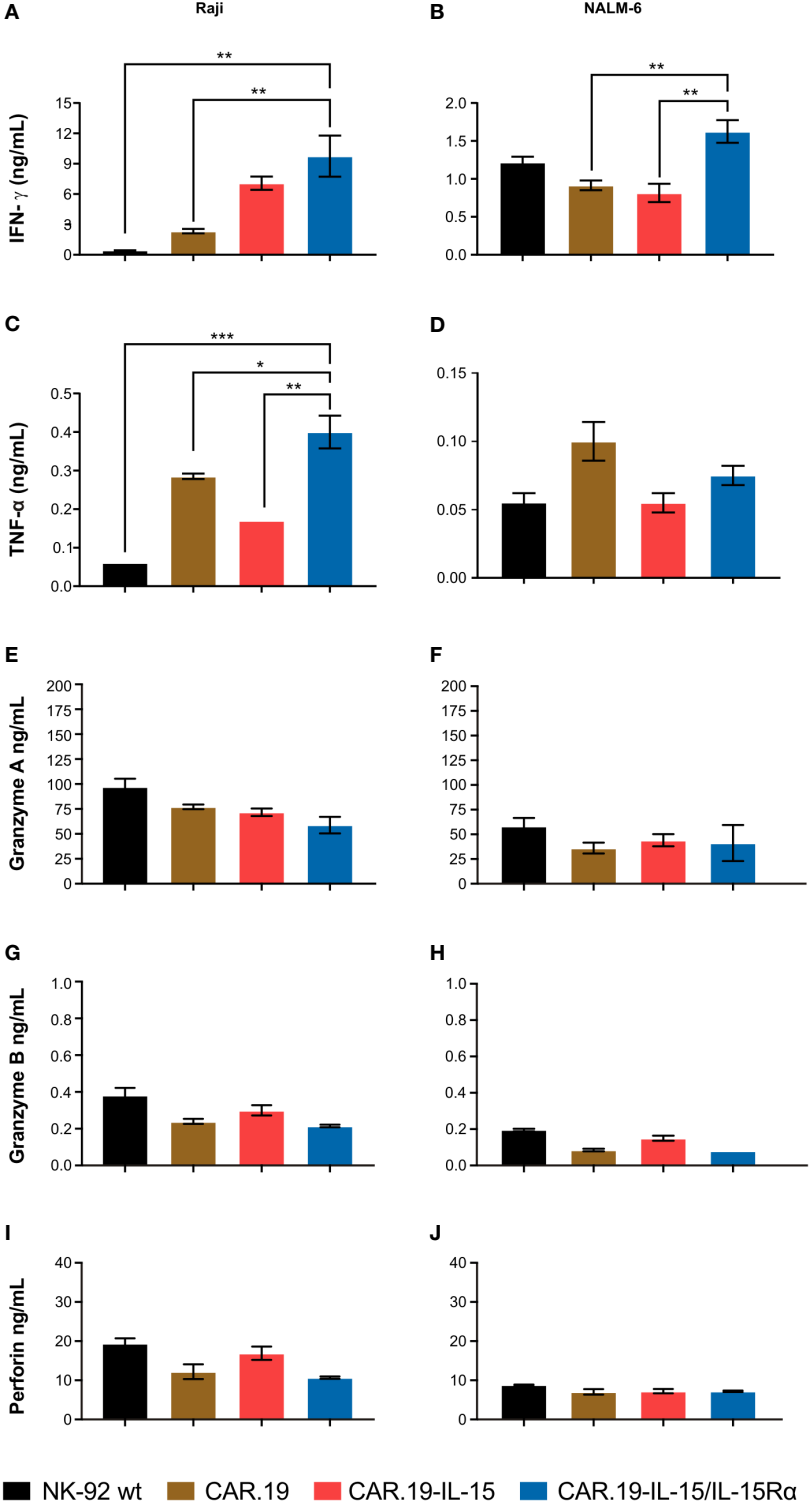
NK-92-CAR.19-IL-15/IL15Rα cells produce high amount of anti-tumor pro-inflammatory cytokines. NK-92, CAR.19, CAR.19-IL-15 and CAR.19-IL-15Rα NK-92 cells (2.5 × 10^5^) were incubated for 5 h with Raji lymphoma cells **(A, C)** or with Nalm-6 **(B, D)** at an E/T ratio of 10:1. Supernatants were collected, and the levels of IFN-γ **(A, B)** and TNF-α **(C, D)**, granzyme A **(E, F)**), granzyme B **(G, H)** and perforin **(I, J)** were measured using the Luminex MAGPIX system. NK cells without target cells were included as controls. One-way ANOVA statistical test, Tukey's multiple comparison posttest. Mean values ± SD are shown; n=2. ***P < 0.001; **P < 0.01; *P < 0.05. Data pooled from three independent experiments.

In order to evaluate the role of IL-15 and IL-15Rα in the production of antitumor cytokines, we co-cultured CAR NK-92 cells with NALM-6 and Raji cells and measured the levels of IFN-γ and TNF-α in supernatants. In contact with Raji cells, second-generation NK-92-CAR.19 cells showed significantly increased release of IFN-γ and TNF-α, which was further enhanced in the case of IL-15-containing CAR (**Figures 4A, C**). NK-92 cells expressing CAR.19-IL-15/IL-15Rα produced the highest levels of IFN-γ after contact with both Raji and NALM-6 cells (**Figures 4A, B**). NK-92-CAR.19-IL-15/IL-15Rα cells also produced more TNF-α after contact with Raji cells and similar levels after contact with NALM-6, compared to controls (**Figures 4C, D**). We also assessed the levels of granzyme A (**Figures 4E, F**), granzyme B (**Figures 4G, H**), and perforin (**Figures 4I, J**), but no significant differences were observed.

### 3.5 Differential gene expression analyses reveal unique pathways upregulated in NK-92 cells expressing IL15/IL15Rα

**Figure 5:**
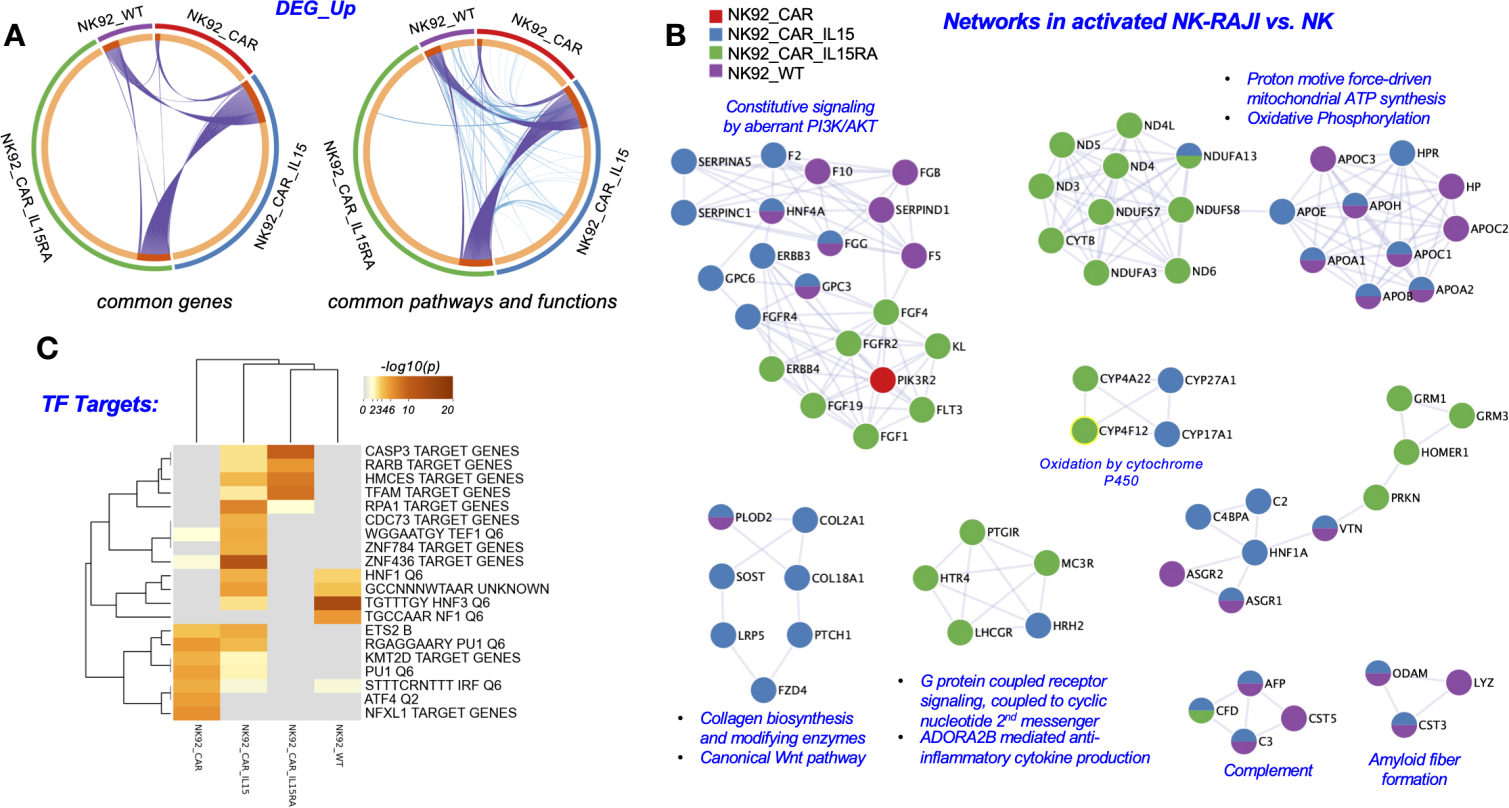
Metascape functional analysis of transcriptome profiles of NK-92-CAR.19 cells after coculture with a target B cell line. **(A)** The Circos plot shows how genes from the different input gene lists after coculture setups overlap. On the outside, the arc represents the identity of each gene list. On the inside, the orange color represents the genes that appear in multiple lists, and the light orange color represents genes that are unique to that gene list. Purple lines link the same genes shared by multiple gene lists. Blue lines link the genes that fall into the same ontology term (the term has to be statistically significantly enriched). The greater the number of purple links and the longer the dark orange arcs, the greater is the overlap among the input gene lists. Blue links indicate the amount of functional overlap among the input gene lists. **(B)** Networks of Protein-Protein interaction (PPI) identified in the gene lists for comparing across NK92-CAR groups and NK92-WT, and illustrated using Cytoscape 3.9.1. **(C)** Summary of enrichment analysis done using the DEG_up from **(A)** and the Trancription_Factor_Targets as an ontology category (Metascape).

**Figure 6:**
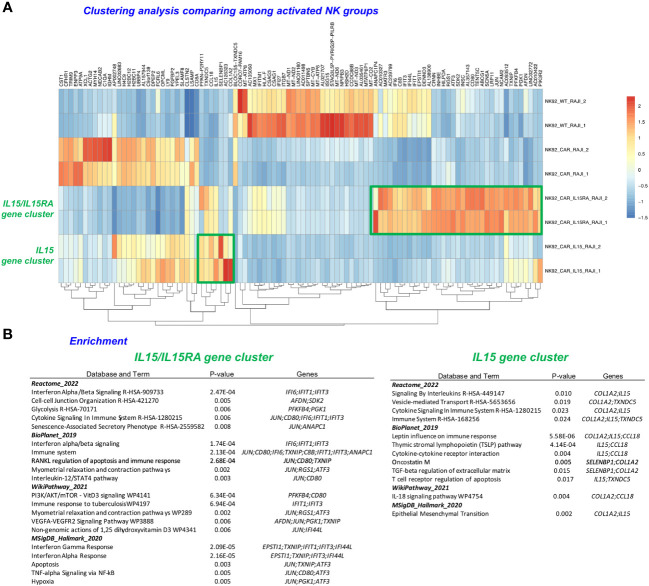
Clustering analysis of the most significantly upregulated and downregulated genes. **(A)** Heatmap of differentially expressed genes (DEGs) upregulated, with clustering of results was done to comparatively illustrate the expression across different samples (IL-15 and IL15/IL15Rα). **(B)** Enrichment analysis using the Enrichr bioinformatics tool following the input of the upregulated (DEG) gene list clusters derived from the DEGs in **(A)** (23).

To elucidate transcriptional signatures of the different NK-92 cells, we conducted bioinformatic analyses of the NK-92-CAR.19 cell variants. We explored the impact of activated NK-92 cells with RAJI cells (NK+RAJI) on NK differential gene expression (**Figure 5**), and also compared across all activated NK to assess mechanisms underlying the higher potency of NK-92-CAR.19-IL-15 and NK-92-CAR.19-IL15/IL-15Rα cells (**Figure 6**).

First, as illustrated in the *circos* plots (**Figure 5A**), we identified a large overlap of genes and biological processes among the different NK-92-CAR.19 cell variants during their activation process. Although the NK groups share many common genes, pathways and functions, the mechanisms of NK activation are different in each NK group. Secondly, additional analyses compared each of the groups in their activated state (NK-92+RAJI) relative to the inactivated state (NK). In a *Metascape* analysis, we show that the mechanisms underlying each groups’ activation had some key differences. For example, all the NK utilize PI3K/AKT related pathways to become activated in the presence of RAJI cells, yet they do not necessarily utilize all the same molecules to achieve the activation. NK-92-CAR.19-IL-15/IL-15Rα cells utilize the FGF1/4/19-FGFR2 and ERBB4 subpathways during activation, and NK-92-CAR.19-IL-15 utilize HNF4A/ERBB3/FGFR4 as they become activated (**Figure 5B**). Moreover, NK-92-CAR.19-IL-15/IL-15Rα cells utilize to a greater extent oxidative phosphorylation pathways, G protein coupled receptors, and anti-inflammatory cytokine production pathways as they become activated, relative to the other NK-CAR cells. Third, we performed a *Transcription Factor Targets* analysis using *Metascape* to identify regulators potentially underlying the different mechanisms of activation for each NK group (**Figure 5C**). This analysis indicated that, when NK-92 WT cells became activated, upregulated DEG were enriched in regulatory elements for HNF1/3 and NF1. For NK-92-CAR, the enrichments were for KMT2D, PU1, IRF, ATF4, and NFXL1, and for NK-92-CAR.19-IL15, the most prominent (-log10(P)) values were for the regulators RPA1, HCMES, and ZNF436, although HNF1/3 were also implicated. For NK-92-CAR.19-IL15/IL-15Rα cells, the most prominent included CASP3 target genes, and genes regulated by RARβ, HMCES, and TFAM.

Lastly, we performed a *clustering analysis* that included the most significantly upregulated and downregulated genes, focusing now only on comparing among the activated NK-CAR cells. The NK-CAR.19-IL-15/IL-15Rα groups had a distinct gene signature that differentiated them not only from parental NK-92, but also from second-generation NK-CAR, as well as from NK-CAR co-expressing the soluble form of IL-15 (**Figure 6A**). Clusters were identified from 34 upregulated genes for NK-92-CAR.19-IL-15/IL-15Rα cells, and from 7 genes for NK-92-CAR.19-IL-15. Further examination of these clusters was performed using the tool *Enrichr* with the databases *Reactome, Bioplanet, WikiPathway*, and *MSigDB-Hallmark*, indicating key pathways and genes and their functions listed in **Figure 6B**, ranked by *p*-value (cutoffs of p<0.01 for the IL-15/IL-15Rα cluster and p<0.03 for the IL-15 cluster). These analyses indicated that the most highly significant pathways and functions underlying the NK-92-CAR-IL15/IL-15Rα higher potency relative to the other NK-CAR include (top 3 per database queried), *IFNα/β signaling*, *Cell-cell junction, Glycolysis, Immune system*, *RANKL regulation of apoptosis and immune response*, *PI3K/AKT/mTOR signaling*, *Immune response to tuberculosis*, *Interferon Gamma response*, and *Apoptosis*. The most significant pathways and functions underlying the NK-92-CAR-IL-15 unique potency (top 2 per database queried) relate to *Signaling by interleukins*, *vesicle-mediated transport*, *Leptin influence on immune response*, *Thymic stromal lymphopoietin pathway*, *cytokine-cytokine receptor interaction*, and the *IL-18 signaling pathway*.

### 3.6 Expression of IL-15 or IL-15/IL-15Rα protects against upregulation of checkpoint receptors in NK-92-CAR.19 cells

**Figure 7:**
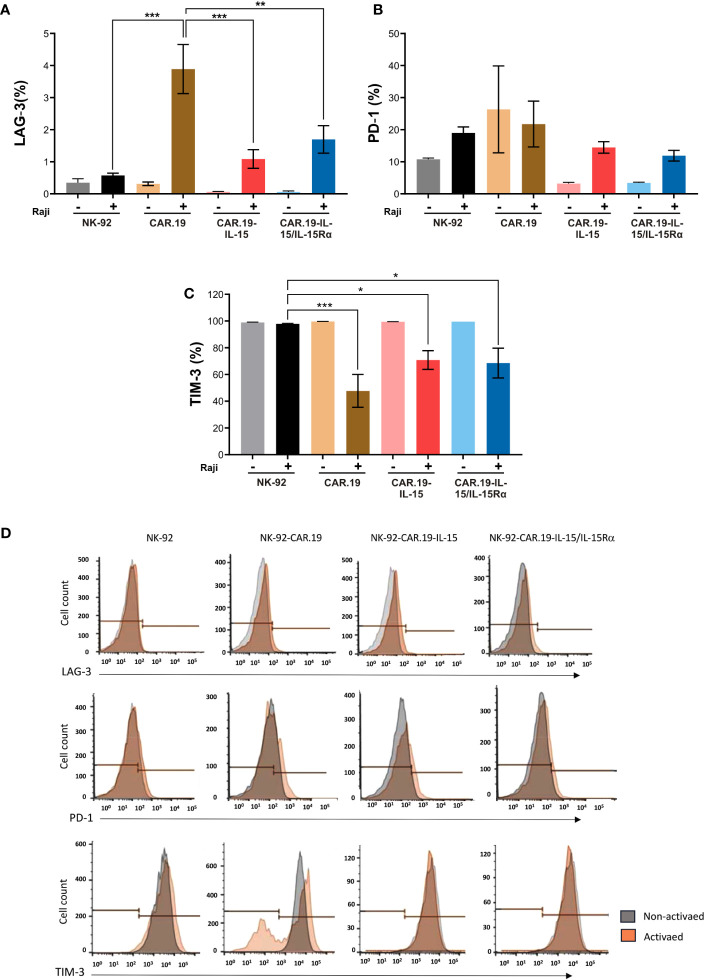
Transgenic IL-15 or IL-15/Rα maintains low expression of immune checkpoint molecules in NK-92-CAR.19 cell variants. NK-92-CAR.19 cells were cultured unstimulated (-) or stimulated (+) with Raji cells at an E:T ratio of 1:2, followed by restimulation with fresh target cells after 24 and 48 hours. Three days after the beginning of the experiment, the surface expression of LAG-3 **(A)**, PD-1 **(B)** and TIM-3 **(C)** were analyzed by flow cytometry. Three independent experiments were performed. One-way ANOVA statistical test, Tukey's *post-hoc* multiple comparison. Mean values + SD are shown. Gray: NK-92, Black: coculture of NK-92 and Raji cells, Orange: CAR.19, Brown: coculture of CAR.19 and Raji cells, Pink: CAR.19-IL-15, Red: cocultured of CAR.19-IL-15 and Raji cells, Light blue: CAR.19-IL-15RA, and Blue: coculture of CAR.19-IL-15 and Raji cells. **(D)** Representative flow cytometry histograms plots showing the mean fluorescence intensity of LAG-3, PD-1 and TIM-3 in NK-92 cell and, in the NK-92-CAR.19 cell variants. ***P < 0.001; **P < 0.01; *P < 0.05.

To assess the effects of repeated stimulation of NK-92-CAR.19 with target CD19+ cells, we co-cultured NK-92-CAR.19, NK-92-CAR.19-IL-15 or NK-92-CAR.19-IL15/IL-15Rα cells with Raji cells for 3 days at an initial E/T ratio of 1:2, and repeated stimulation by adding fresh target cells after 24 and 48 h (**Figure 7**). Surface expression of PD-1, LAG-3 and TIM-3, which are inhibitory immune receptors associated with an exhaustion of lymphocytes (24), was examined by flow cytometry and is shown as the percentage of NK-92 cells expressing this parameter. The expression of these markers in the restimulated NK-92 cells was determined by flow cytometry and compared with unstimulated NK-92-CAR cells.

The expression of PD-1 and LAG-3 increased after repeated stimulation in all engineered NK-92-CAR cells (**Figures 7A, B**), albeit at low levels. Interestingly, Raji-stimulated NK-92-CAR.19 variants expressing either IL-15 or IL-15/IL-15Rα showed overall reduced levels of LAG-3 and PD-1 expression compared to stimulated NK-92-CAR.19, possibly reflecting better fitness and less exhaustion due to IL-15 signaling. Interestingly, all NK-92 variants fully expressed TIM-3, which was significantly reduced in CAR-NK cells after stimulation with CD19^+^ cells, while remaining unchained in parental NK-92, suggesting that CAR activation leads to TIM-3 downregulation (**Figure 7C**).

### 3.7 NK-92-CAR.19-IL-15/IL-15Rα enhanced *in vivo* cytotoxic function against B cell lymphoma

**Figure 8:**
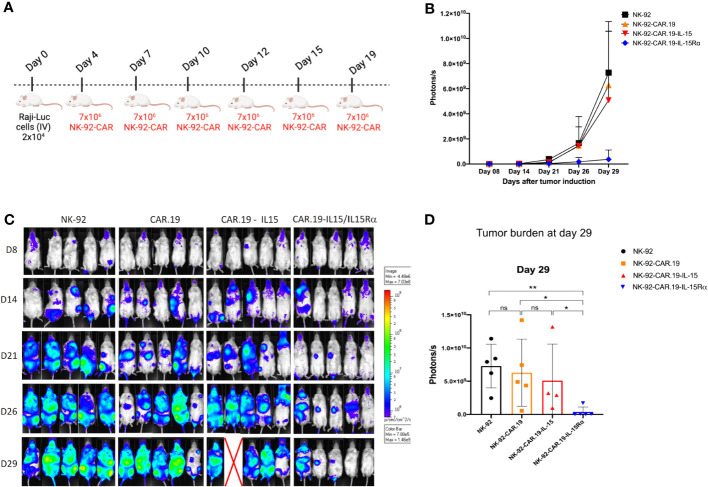
NK-92-CAR.19-IL15/IL-15Rα cells eliminate lymphoma B-cells and reduce tumor burden *in vivo*. **(A)** Scheme of an *in vivo* study demonstrating the anti-tumor activity of transduced NK-92-CAR.19 cells in a xenogeneic NSG mouse model of disseminated human B-cell hematologic malignancy. NSG mice were intravenously injected with 2 × 10^4^ Raji-Luc lymphoma B-cells. On days 4, 7, 10, 12, 15, and 19, animals were treated by intravenous injection of 7 × 10^6^ NK-92 cells (n=5). **(B)** Lymphoma development was monitored by *in vivo* bioluminescence imaging. Images were taken on days 8, 14, 21, 26, and 29. **(C)** Bioluminescence intensity was quantified and means + SEM are shown. **(D)** Bioluminescence of individual mice shown on day 29. Means + SEM are shown. Statistics were determined by the Mann-Whitney test. Each experimental group consisted of five mice. **P < 0.01; *P < 0.05.

We, finally, challenged the anti-lymphoma activity of NK-92-CAR.19-IL-15/IL-15Rα cells in a Raji xenograft immunodeficient mouse model. Mice received one intravenous infusion (2x 10^4^/mouse) of Raji-Luc^+^ cell line (D0) followed by six infusions of NK-92-CAR.19-IL15/IL-15Rα, NK-92-CAR.19-IL15, NK-92-CAR.19 or parental NK-92 cells on days 4, 7, 10, 12, 15, and 19 (7x10^6^/mouse) (**Figure 8A**). Tumor growth was monitored by measuring bioluminescence over time. As expected, bioluminescence increased rapidly in mice treated with control NK-92 cells (**Figures 8B, C**). NK-92 cells expressing CAR.CD19 or CAR.19-IL-15 slowed tumor progression until day 21, but relapsed at later time points (**Figures 8C, D**). In contrast, NK-92-CAR.19-IL15/IL-15Rα cells resulted in tumor growth control for the entire 29 days. Thus, CD19.CAR NK cells co-expressing IL15/IL15Rα exhibited superior antitumor activity against lymphoma cells *in vivo* compared to CAR construct co-expressing soluble IL-15 or no cytokines.

## 4 Discussion

The clinical use of off-the-shelf therapeutic NK-92 cells may help more patients to get access to NK-CAR cell-based therapy due to logistics and costs (25). In our research study, we used the NK-92 cell line due to its ease of manipulation and high cytotoxic potential against cancer cells when combined with CAR technology. Several *in vitro* studies and preclinical models have shown the effectiveness of CAR-NK-92 cells against a variety of cancers, including hematological (26) and solid tumors (26–28). In fact, armoring NK-92 with targeted CARs has been shown to overcome tumor inhibitory signals (29) and confer sensibility of target cells towards NK mediated killing by signaling activation through CAR. Therefore, these cells have become an attractive option for clinical translation, as a truly off-the-shelf therapeutic cell-based therapy with a great expansion capacity (29). At present, only few clinical trials using CAR-engineered NK cells are ongoing and despite all the attention, the clinical development of CAR-NK cells is years behind that of CAR-T cells, which have already gained market authorizations for many hematologic malignancies. Beside technical hurdles in the genetic manipulation, expansion and cryopreservation of NK cells, which may account for the delay in clinical development, NK cells and CAR-NK cells have thus far failed to show convincing clinical benefit, as compared to CAR-T cells. This may in part also be attributed to the inability of NK cells to expand *in vivo* and to reach favorable effector to target ratios in a clinical setting.

IL-15 is an essential cytokine for NK development, survival, proliferation, and function (30). Different functional forms of IL-15 exist in humans comprising soluble IL-15 and also membrane bound IL-15/IL-15Rα complexes have been described and it remains to be determined, which form may be best suited to be used within CAR-engineered NK cells. Soluble IL-15 is currently the most common form incorporated in engineered CAR-NK cells used in preclinical and clinical studies (3, 24).

In this work, we have engineered an innovative vector expressing IL-15 linked to its high affinity receptor alpha IL-15Rα via a flexible SG-link, inspired by the natural trans-presentation of IL-15, in combination with a CAR construct to enhance the cytotoxicity potential of NK-92 cells towards CD19+ target cells.

We showed that NK-92-CAR.19 cells expressing IL-15 or IL-15/IL-15Rα significantly enhanced *in vitro* antitumor activity compared to cells without transgenic cytokines. Although the CAR.19 construct could also mediate an antitumor response, soluble IL-15 or IL-15/IL-15Rα improved the cytotoxicity potential of second-generation NK-92.CAR.19 cells. The high cytotoxicity may in part be explained by the intrinsic and continuous supply of IL-15. However, recombinant expression of IL-15 also shaped the level of important cytotoxic cytokines that are released from NK cells. When analyzing the cytokine profiles of the different NK-92 variants, cells transduced with a CAR that also expresses IL-15 showed increased levels of cytokine release. Notably, NK-92-CAR.19-IL-15/IL-15Rα were shown to secrete the highest amounts of TNF-α and IFN-γ upon activation, which suggested that part of the enhanced cytotoxicity may be attributed to these cytokines. These findings are in line with recent findings that also highlighted the beneficial effect of IL-15 on NK cells regarding antitumor activity (31–34). In fact, Conlon et al. described a massive expansion of NK cells in patients who received continuous infusions of IL-15. Furthermore, IL-15 treatment resulted in increased secretion of granzymes A and B and perforin in NK cells, indicating that IL-15 infusions may potentiate their cytotoxic antitumor response (32). It has been shown recently that improper polarization of lytic granules towards immunological synapses in NK cells, following contact with resistant cancers, leads to inefficient cytotoxicity. It has been also shown that soluble IL-2 can increase lytic granule convergence (35). Given the fact that IL-2 and IL-15 use the same receptor to activate NK cells, one could hypothesize that IL-15 is likely to also have a positive role on the granule machinery and that IL-15 or IL-15/IL-15Rα in the engineered CAR-NK may have facilitated polarization of the lytic granule machinery to the immunological synapse. Further studies are required to demonstrate whether IL-15/IL-15Rα could induce stronger or faster convergence or polarization of lytic granules in CAR-NK cells.

In order to unravel genes and/or pathways expressed by the soluble and transmembrane forms of IL-15 signaling, RNA sequencing was performed on the activated NK-92-CAR.19 cells. Bioinformatic analysis demonstrated that NK-92-CAR.19-IL-15 and NK-92-CAR.19-IL-15/IL-15Rα cells had distinct gene expression profiles. Both soluble IL-15 and IL-15/IL-15Ra triggered a cascade of intracellular events that ultimately activated the PI3K/AKT and the JAK/STAT signaling pathways; however, by recruiting different players. The PI3K/AKT signaling pathway is one of the well-described mechanisms promoting NK survival, proliferation, and their effector functions (23). Furthermore, additional analyses suggested that specific regulators and pathways might underlie the higher potency of NK-92-CAR-IL15/IL15Rα. In particular, clustering analyses suggested that NK-92-CAR-IL15/IL15Rα significantly upregulated genes in the interferon type I and II pathways, PI3K/AKT/mTOR pathway, and also metabolic functions, such as enhanced glycolysis, and regulation of apoptosis and immune response.

The PI3K/AKT pathway plays a critical role in cellular signaling, governing essential processes such as cell survival, proliferation, growth, and metabolism. This pathway is connected with immune functions, particularly those involving NK cells (36). Our analysis has identified a significant network associating a gene set with a strong link to both the PI3K/AKT pathway and NK cell activity. Among these genes, FGF4, a fibroblast growth factor, indirectly influences the PI3K/AKT pathway through receptor tyrosine kinases. FGFR2, an FGF receptor, activates downstream pathways, including PI3K/AKT, impacting cell survival and the immune functions of NK cells. Additionally, ERBB4, a member of the EGFR family, activates the PI3K/AKT pathway, influencing cell survival, proliferation, and various immune cell functions, including those of NK cells. FLT3, a receptor kinase involved in immune cell development, can activate PI3K/AKT, affecting cell growth and immune cell function. FGF19 also activates the PI3K/AKT pathway, establishing a link between metabolism, immune responses, and NK cell functions. KL, which encodes an anti-aging protein, interacts with the PI3K/AKT pathway through metabolic pathways, potentially impacting immune cell functions as well.

Furthermore, the PI3K/AKT pathway enhances NK cell cytotoxicity by influencing granule exocytosis, target recognition, adhesion, and the regulation of key cytotoxic molecules (37). This pathway also plays a role in NK cell migration and activation, which underlies the observed increase in target cell killing. Moreover, the PI3K/AKT pathway could potentially reduce the dependency on IL-2 for NK cell proliferation and survival. Therefore, activating the PI3K/AKT pathway not only enhances cytotoxicity but also facilitates NK cell growth, resulting in a larger pool of cytotoxic NK cells ready for immune responses. This strategy has the potential to decrease the need for high doses of IL-2, potentially revolutionizing NK cell-based therapies. Additionally, our study revealed several genes related to mitochondrial metabolism, such as NADH dehydrogenase subunits (ND3, ND4, ND5, ND6), among others. Future investigations could focus on examining the impact of gene family knockdown (FGF/FGFR or ND) using techniques like siRNA or CRISPR on the response of NK-CAR-IL15RA cells in culture, both in the presence and absence of IL-2.”

PD-1, LAG-3 and TIM-3 are molecules associated with inhibitory activity or exhaustion in NK cells and other lymphocytes (38). Although we have found comparable levels of these checkpoint molecules in unstimulated NK-92-CAR.19 cells, repeated stimulation with CD19+ target cells differentially affected the expression of these inhibitory molecules on the NK-92-CAR.19 cell variants. Most strikingly, PD-1 and LAG-3 expression was reduced in NK-92-CAR.19-IL-15 and NK-92-CAR.19-IL-15/IL-15Rα as compared with NK-92-CAR.19 cells. Therefore, our data suggest that NK-92-CAR.19 cells producing IL-15 or IL-15/IL-15Rα might be more robust and less sensitive to regulation by checkpoint inhibition.

Our data further suggest that IL-15/IL-15Rα is sufficient to confer enhanced proliferation even without exogenous IL-2. In fact, our experiments showed that only IL-15/IL-15Rα allowed IL-2 independent proliferation, whereas soluble IL-15 did not. NK-92-CAR.19 cells expressing soluble IL-15 proliferated only for 9 to 12 days in culture without IL-2.

To analyze if the beneficial results for IL-15 carrying CARs would also translate into potentially clinical meaningful results in an *in vivo* model, we compared all NK-92 variants in a xenograft NSG mice model against CD19+ Raji lymphoma cells. Neither parental NK-92 cells nor NK-92-CAR cells have thus far been reported to proliferate *in vivo* and/or to permanently engraft in NSG mice, under the current experimental setting of intravenous application (24). So far, in clinical setting, NK-92 have always been irradiated to prevent them from proliferation. For this reason, we have used a systemic injection scheme of NK cells, with repeated intravenous injections in mice. Treatment with parental NK-92 cells had little effect, resulting in extensive growth of disseminated lymphoma in several organs. In mice treated with CAR.19 and CAR.19-IL-15 NK-92 cells, the growth of Raji cells seemed to be delayed on day 21, respectively, but at day 26 the mice showed comparable high tumor cell dissemination and load compared to mice treated with parental NK-92 cells. On the contrary, it was solely the CAR.19 NK-92 cells expressing the IL-15/IL-15Rα construct that demonstrated the ability to regulate tumor progression. This specific construct showcased its potential to augment NK-92 cell activation within the mice, resulting in successful tumor control. We suggest that the increased secretion potential of TNF-α and IFN-γ observed in the *in vitro* experiments may also have contributed to this effect. Our data demonstrated that IL-15/IL-15Rα is a better alternative than soluble transgenic IL-15 secretion by second-generation NK-92-CAR cells. In a recent study, the team led by Rezvani demonstrated that compared to NK cells and NK cells expressing CAR19 alone, armoring CAR19 NK cells with soluble IL-15 resulted in a remarkable increase in their proliferation rate and effector function (39).

**Figure 9:**
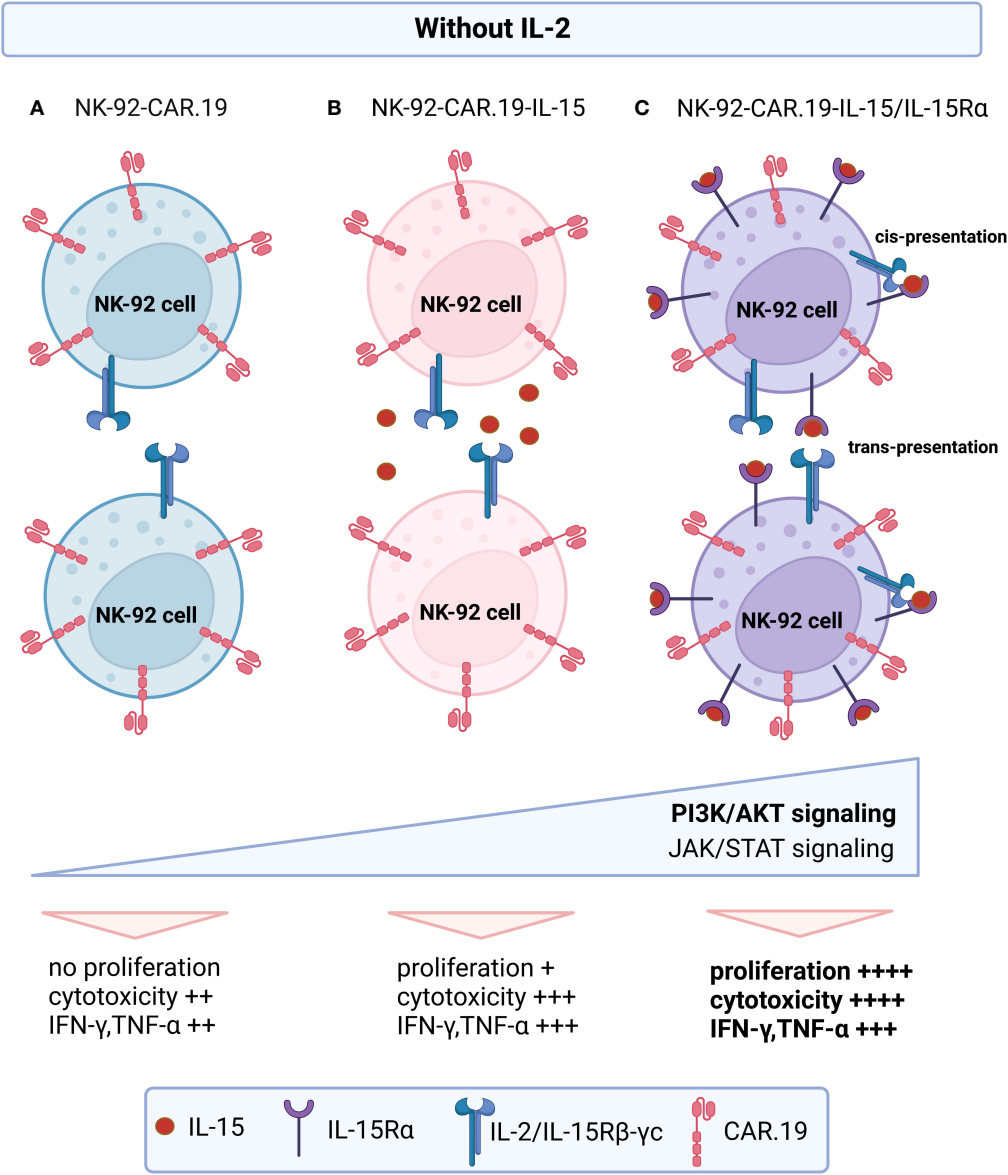
Proposed model of action of engineered NK-92-CAR-19-IL-15/IL-15Rα cells. In the absence of exogenous IL-2: **(A)** NK-92-CAR.19 cells remain non-proliferative. **(B)** NK-92-CAR.19-IL-15 cells exhibit modest proliferation up to day 12, as they release soluble IL-15 that subsequently engages with IL-2/IL-15Rβ–γc receptors on their own membrane or neighboring NK cells. **(C)** NK-92-CAR.19-IL-15/IL-15Rα cells display robust proliferation, driven by the presence of IL-15/IL-15Rα complexes on their cell membrane. This activates the cells through cis-presentation or may stimulate adjacent NK cells expressing membrane-bound IL-2/IL-15Rβ–γc receptors via trans-presentation. The signaling from IL-15 prompts all three NK-92-CAR-19 cell variants to upregulate the PI3K/AKT and JAK/STAT pathways. However, this upregulation is notably more pronounced in NK-92-CAR.19-IL-15/IL-15Rα cells compared to the other variants. Consequently, this substantial enhancement in signaling leads to heightened proliferative capacity, increased secretion of proinflammatory cytokines, and exceptionally potent cytotoxic activity in these pioneering engineered NK-92 cells.

In conclusion, we described here a novel immunotherapy approach using engineered NK-92-CAR.19 cells that express IL-15 linked to its receptor IL-15Rα, with strong cytotoxic activity, high secretion of proinflammatory cytokines, which can be highly expanded independently of IL-2 (**Figure 9**). All these features favor large-scale NK cell production for clinical applications with lower costs and improved therapeutic potency. Our data highlighted the critical nature of IL-15/IL-15Rα complex signaling as a mechanism that can enhance *in vitro* NK cell expansion along with target-specific cytotoxic function of NK-CAR cells. The *in vivo* results demonstrated the enhanced anti-tumor potential of NK-92-CAR-19-IL-15/IL-15Rα cells, which may further improve the clinical use of NK-CAR cells. Although we have chosen CD19 as target for this proof-of-concept study, other targets may be used with this innovative vector construct, thereby extending the therapeutic application of this NK-CAR cell platform to other cancer types.

## Data availability statement

The original contributions presented in the study are publicly available. This data can be found here: https://www.ncbi.nlm.nih.gov/geo/query/acc.cgi?acc=GSE234733.

## Ethics statement

Ethical approval was not required for the studies on humans in accordance with the local legislation and institutional requirements because only commercially available established cell lines were used. The animal study was approved by CEUA government committee in Brazil (n. 124/2017). The study was conducted in accordance with the local legislation and institutional requirements.

## Author contributions

RS performed the experiments and drafted the original manuscript and figures. JE performed the experiments and drafted the original manuscript and figures. JA helped with some experiments and drafted the original manuscript and figures. MT helped with some experiments and drafted the original manuscript. DF helped with *in vivo* assays. LB helped with *in vivo* assays. KM drafted and edited the manuscript. MF helped with analysis of the RNAseq data. KS drafted and edited the original manuscript. RC analyzed the data and edited the manuscript. PM edited the original manuscript, DC edited the original manuscript. TT drafted the original manuscript and figures and VP-C analyzed the data, drafted the original manuscript and figures. All authors contributed to the article and approved the submitted version.

## References

[1] Suck G Odendahl M Nowakowska P Seidl C Wels WS Klingemann HG. NK-92: an ‘off-the-shelf therapeutic’ for adoptive natural killer cell-based cancer immunotherapy. Cancer Immunol. Immunother. (2016) 65:485–92. doi: 10.1007/s00262-015-1761-x PMC11029582 26559813

[2] Klingemann H. Are natural killer cells superior CAR drivers? Oncoimmunology (2014) 3. doi: 10.4161/onci.28147 PMC4203506 25340009

[3] Liu E Marin D Banerjee P Macapinlac HA Thompson P Basar R. Use of CAR-transduced natural killer cells in CD19-positive lymphoid tumors. New Engl J Med (2020) 382:545–53. doi: 10.1056/NEJMOA1910607 PMC7101242 32023374

[4] Liu E Tong Y Dotti G Shaim H Savoldo B Mukherjee M. Cord blood NK cells engineered to express IL-15 and a CD19-targeted CAR show long-term persistence and potent antitumor activity. Leukemia (2018) 32:520–31. doi: 10.1038/leu.2017.226 PMC6063081 28725044

[5] Waldmann TA Tagaya Y. The multifaceted regulation of interleukin-15 expression and the role of this cytokine in NK cells differentiation and host response to intracellular pathogens. Annu Rev Immunol (1999) 17:19–49. doi: 10.1146/annurev.immunol.17.1.19 10358752

[6] Bergamaschi C Bear J Rosati M Beach RK Alicea C Sowder R. Circulating IL-15 exists as heterodimeric complex with soluble IL-15Rα in human and mouse serum. Blood (2012) 120:e1–8. doi: 10.1182/blood-2011-10-384362 PMC3390963 22496150

[7] Burkett PR Koka R Chien M Chai S Boone DL Ma A. Coordinate expression and trans presentation of interleukin (IL)-15Rα and IL-15 supports natural killer cell and memory CD8+ T cell homeostasis. J Exp Med (2004) 200:825–34. doi: 10.1084/jem.20041389 PMC2213280 15452177

[8] Giron-Michel J Giuliani M Fogli M Brouty-Boyé D Ferrini S Baychelier F. Membrane-bound and soluble IL-15/IL-15Rα complexes display differential signaling and functions on human hematopoietic progenitors. Blood (2005) 106:2302–10. doi: 10.1182/blood-2005-01-0064 15976182

[9] Waldmann TA. The biology of interleukin-2 and interleukin-15: implications for cancer therapy and vaccine design. Nat Rev Immunol (2006) 6:595–601. doi: 10.1038/nri1901 16868550

[10] Neely GG Epelman S Ling Ma L Colarusso P Howlett CJ Amankwah EK. Monocyte surface-bound IL-15 can function as an activating receptor and participate in reverse signaling. J Immunol (Baltimore Md.: 1950) (2004) 172(7):4225–34. doi: 10.4049/jimmunol.172.7.4225 15034035

[11] lodolce JP Burkett PR Koka RM Boone DL Ma A. Regulation of lymphoid homeostasis by interleukin-15. Cytokine Growth Factor Rev (2002) 13(6):429–39. doi: 10.1016/s1359-6101(02)00029-1 12401478

[12] Schluns KS Stoklasek T Lefrançois L. The roles of interleukin-15 receptor α: Trans-presentation, receptor component, or both? Int J Biochem Cell Biol (2005) 37(8):1567–71. doi: 10.1016/j.biocel.2005.02.017 15896666

[13] Han K Zhu X Liu B Jeng E Kong L Yovandich JL. IL-15:IL-15 receptor alpha superagonist complex: High-level co-expression in recombinant mammalian cells, purification and characterization. Cytokine (2011) 56:804–10. doi: 10.1016/j.cyto.2011.09.028 PMC3221918 22019703

[14] Chertova E Bergamaschi C Chertov O Sowder R Bear J Roser JD. Characterization and favorable *in vivo* properties of heterodimeric soluble IL-15·IL-15Rα Cytokine compared to IL-15 monomer*. J Biol Chem (2013) 288:18093–103. doi: 10.1074/jbc.M113.461756 PMC3689953 23649624

[15] Rubinstein MP Williams C Mart C Beall J MacPherson L Azar J. Phase I trial characterizing the pharmacokinetic profile of N-803, a chimeric IL-15 superagonist, in healthy volunteers. J Immunol (2022) 208:1362–70. doi: 10.4049/jimmunol.2100066 35228263

[16] Anton OM Peterson ME Hollander MJ Dorward DW Arora G Traba J. Trans-endocytosis of intact IL-15Rα–IL-15 complex from presenting cells into NK cells favors signaling for proliferation. Proc Natl Acad Sci (2020) 117:522–31. doi: 10.1073/pnas.1911678117 PMC6955382 31871169

[17] Gong JH Maki G Klingemann HG. Characterization of a human cell line (NK-92) with phenotypical and functional characteristics of activated natural killer cells. Leukemia (1994) 8(4):652–8. 8152260

[18] Tonn T Becker S Esser R Schwabe D Seifried E. Cellular immunotherapy of Malignancies using the clonal natural killer cell line NK-92. J Hematother Stem Cell Res (2001) 10:535–44. doi: 10.1089/15258160152509145 11522236

[19] Nowakowska P Romanski A Miller N Odendahl M Bonig H Zhang C. Clinical grade manufacturing of genetically modified, CAR-expressing NK-92 cells for the treatment of ErbB2-positive Malignancies. Cancer Immunol. Immunother. (2018) 67:25–38. doi: 10.1007/s00262-017-2055-2 28879551 PMC11028154

[20] Picanço-Castro V Moço PD Mizukami A Vaz LD de Souza Fernandes Pereira M Silvestre RN. Establishment of a simple and efficient platform for car-t cell generation and expansion: from lentiviral production to *in vivo* studies. Hematol Transfus Cell Ther (2020) 42:150–8. doi: 10.1016/j.htct.2019.06.007 PMC7248496 31676276

[21] Mortier E Quéméner A Vusio P Lorenzen I Boublik Y Grötzinger J. Soluble interleukin-15 receptor α (IL-15Rα)-sushi as a selective and potent agonist of IL-15 action through IL-15Rβ/γ. J Biol Chem (2006) 281:1612–9. doi: 10.1074/jbc.M508624200 16284400

[22] Figueiredo ML Letteri R Chan-Seng D Kumar S Rivera-Cruz CM Emrick TS. Reengineering tumor microenvironment with sequential interleukin delivery. Bioengineering (2021) 8:90. doi: 10.3390/bioengineering8070090 34209203 PMC8301035

[23] Ali AK Nandagopal N Lee S-H. IL-15–PI3K–AKT–mTOR: A critical pathway in the life journey of natural killer cells. Front Immunol (2015) 6:355. doi: 10.3389/fimmu.2015.00355 26257729 PMC4507451

[24] Anderson AC Joller N Kuchroo VK. Lag-3, tim-3, and TIGIT: co-inhibitory receptors with specialized functions in immune regulation. Immunity (2016) 44:989–1004. doi: 10.1016/j.immuni.2016.05.001 27192565 PMC4942846

[25] Klingemann H Boissel L Toneguzzo F. Natural killer cells for immunotherapy - advantages of the NK-92 cell line over blood NK cells. Front Immunol (2016) 7:91. doi: 10.3389/fimmu.2016.00091 27014270 PMC4789404

[26] Romanski A Uherek C Bug G Seifried E Klingemann H Wels WS. CD19-CAR engineered NK-92 cells are sufficient to overcome NK cell resistance in B-cell Malignancies. J Cell Mol Med (2016) 20:1287–94. doi: 10.1111/jcmm.12810 PMC4929308 27008316

[27] Mitwasi N Feldmann A Arndt C Koristka S Berndt N Jureczek J. “UniCAR”-modified off-the-shelf NK-92 cells for targeting of GD2-expressing tumor cells. Sci Rep (2020) 10:2141. doi: 10.1038/s41598-020-59082-4 32034289 PMC7005792

[28] Schönfeld K Sahm C Zhang C Naundorf S Brendel C Odendahl M. Selective inhibition of tumor growth by clonal NK cells expressing an erbB2/HER2-specific chimeric antigen receptor. Mol Ther (2015) 23:330–8. doi: 10.1038/mt.2014.219 PMC4445620 25373520

[29] Eitler J Wotschel N Miller N Boissel L Klingemann HG Wels W. Inability of granule polarization by NK cells defines tumor resistance and can be overcome by CAR or ADCC mediated targeting. J Immunother Cancer (2021) 9. doi: 10.1136/jitc-2020-001334 PMC7817806 33468562

[30] Becker PSA Suck G Nowakowska P Ullrich E Seifried E Bader P. Selection and expansion of natural killer cells for NK cell-based immunotherapy. Cancer Immunol Immunother (2016) 65:477–84. doi: 10.1007/s00262-016-1792-y PMC4826432 26810567

[31] Teng K-Y Mansour AG Zhu Z Li Z Tian L Ma S. Off-the-shelf prostate stem cell antigen–directed chimeric antigen receptor natural killer cell therapy to treat pancreatic cancer. Gastroenterology (2022) 162:1319–33. doi: 10.1053/j.gastro.2021.12.281 PMC8963130 34999097

[32] Conlon KC Potter EL Pittaluga S Lee C-CR Miljkovic MD Fleisher TA. IL15 by continuous intravenous infusion to adult patients with solid tumors in a phase I trial induced dramatic NK-cell subset expansion. Clin Cancer Res (2019) 25:4945–54. doi: 10.1158/1078-0432.CCR-18-3468 PMC6697593 31142503

[33] Dubois S Conlon KC Müller JR Hsu-Albert J Beltran N Bryant BR. IL15 infusion of cancer patients expands the subpopulation of cytotoxic CD56bright NK cells and increases NK-cell cytokine release capabilities. Cancer Immunol Res (2017) 5:929–38. doi: 10.1158/2326-6066.CIR-17-0279 PMC8177006 28842470

[34] Wagner JA Rosario M Romee R Berrien-Elliott MM Schneider SE Leong JW. CD56bright NK cells exhibit potent antitumor responses following IL-15 priming. J Clin Invest (2017) 127:4042–58. doi: 10.1172/JCI90387 PMC5663359 28972539

[35] James AM Hsu H-T Dongre P Uzel G Mace EM Banerjee PP. Rapid activation receptor– or IL-2–induced lytic granule convergence in human natural killer cells requires Src, but not downstream signaling. Blood (2013) 121:2627–37. doi: 10.1182/blood-2012-06-437012 PMC3617630 23380740

[36] Guo H Samarakoon A Vanhaesebroeck B Malarkannan S. The p110δ of PI3K plays a critical role in NK cell terminal maturation and cytokine/chemokine generation. J Exp Med (2008) 205(10):2419–35. doi: 10.1084/jem.20072327 PMC2556795 18809712

[37] Mace EM. Phosphoinositide-3-kinase signaling in human natural killer cells: new insights from primary immunodeficiency. Front Immunol (2018) 9:445. doi: 10.3389/fimmu.2018.00445 29563913 PMC5845875

[38] Tarazona R Sanchez-Correa B Casas-Avilés I Campos C Pera A Morgado S. Immunosenescence: limitations of natural killer cell-based cancer immunotherapy. Cancer Immunol. Immunother. (2017) 66:233–45. doi: 10.1007/s00262-016-1882-x PMC11029053 27530271

[39] Li L Mohanty V Dou J Huang Y Banerjee PP Miao Q. Loss of metabolic fitness drives tumor resistance after CAR-NK cell therapy and can be overcome by cytokine engineering. Sci Adv (2023) 9(30):eadd6997. doi: 10.1126/sciadv.add6997 37494448 PMC10371011

