# Supplementary material for: Engineering NK-CAR.19 cells with the IL-15/IL-15Rα complex improved proliferation and anti-tumor effect in vivo
Source: Front Immunol. 2023 Sep 25;14:1226518. doi: 10.3389/fimmu.2023.1226518 (PMC10561086; doi:10.3389/fimmu.2023.1226518)
Supplement: Supplementary file 1 [file DataSheet_1.docx]

Supplementary Material


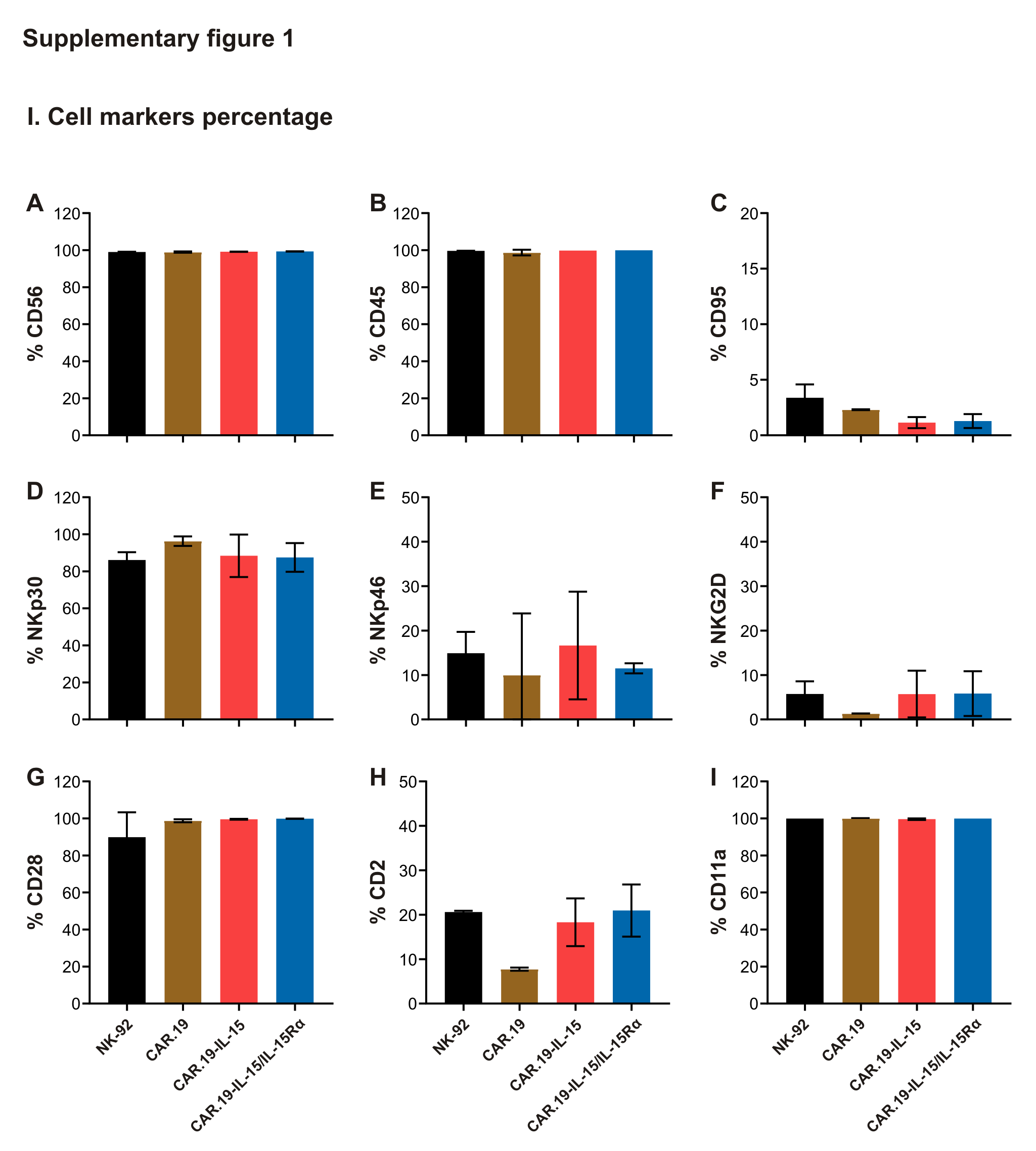


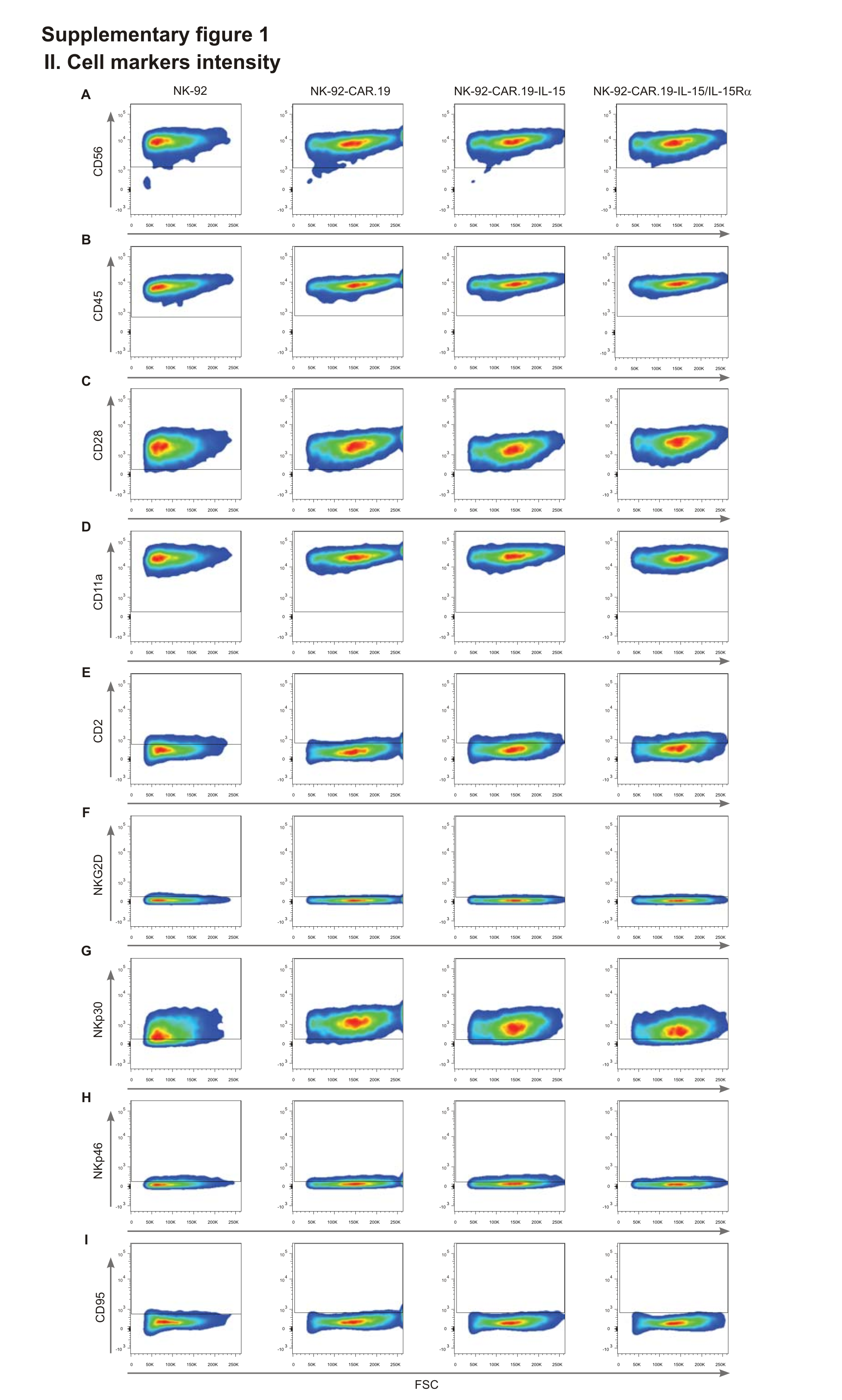


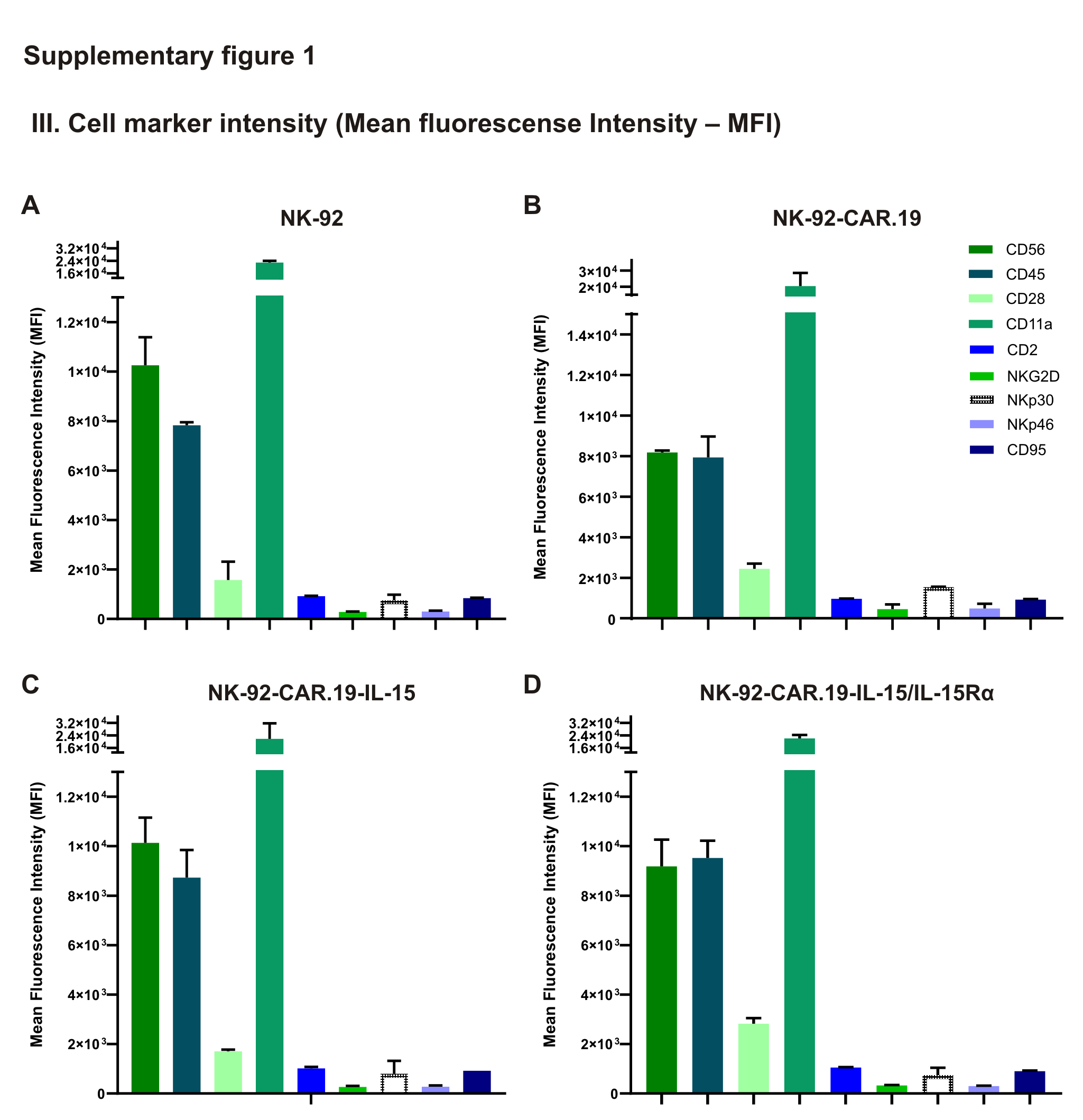


**Supplemental Figure 1. Immunophenotyping of NK-92-CAR.19 cells.** The graphs are displaying the expression levels of the indicated surface markers in different CAR-NK cells and the parental NK-92 cell line. The data shown is a pooled result of two independent experiments.

**Supplementary Figure 2**


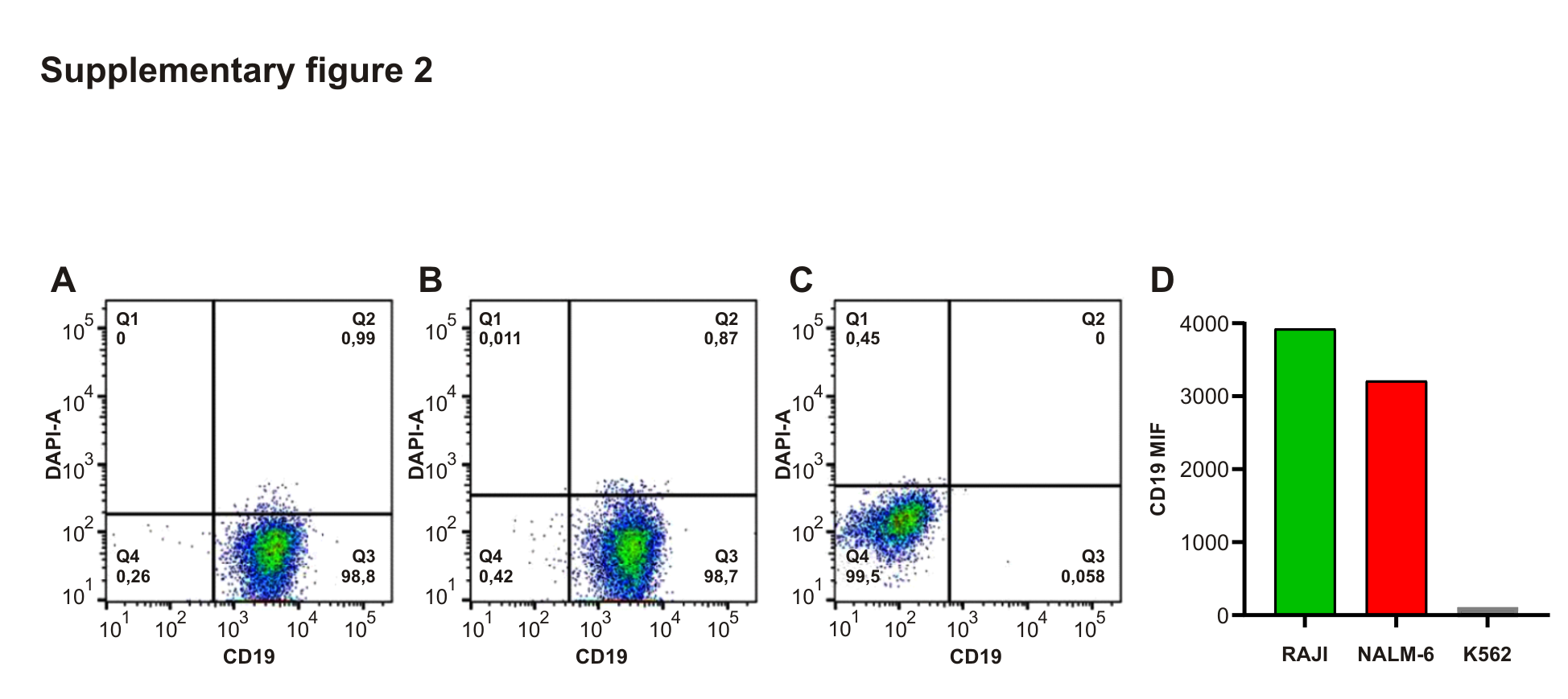


**Supplemental Figure 2.** CD19 expression levels in Raji, Nalm-6 and K562. (A) Raji cell line, (B) Nalm-6 and (C) K562 cell line. Target cell lines were assessed by flow cytometry. The percentage of positive cells (indicated by Q2 and Q3) and the MFI (D) are shown.
